# DIETARY CARBOHYDRATE QUALITY IN RELATION TO HEALTHY AGING IN WOMEN

**DOI:** 10.1093/geroni/igad104.0290

**Published:** 2023-12-21

**Authors:** Andres Ardisson Korat, Kyla Shea, Paul Jacques, Paola Sebastiani, Molin Wang, Walter Willett, Qi Sun

**Affiliations:** Jean Mayer USDA Human Nutrition Research Center on Aging at Tufts University , Boston, Massachusetts, United States; Tufts University, Boston, Massachusetts, United States; Tufts University, Boston, Massachusetts, United States; Tufts Medical Center, Boston, Massachusetts, United States; Harvard T.H. Chan School of Public Health, Boston, Massachusetts, United States; Harvard T.H. Chan School of Public Health, Boston, Massachusetts, United States; Harvard T.H. Chan School of Public Health, Boston, Massachusetts, United States

## Abstract

Our objective was to evaluate the role of dietary carbohydrate quality in healthy aging among women in the Nurses’ Health Study (NHS). We included 29,635 NHS participants aged <60 years old in 1984. Total carbohydrate, refined carbohydrate, dietary glycemic index (GI), dietary fiber intake and carbohydrate from fruits, vegetables, whole and refined grains were derived from validated food-frequency questionnaires. Healthy aging was defined as being free from major chronic diseases, having good mental health, and not having any impairments in cognitive or physical function as assessed in the 2014 or 2016 participant questionnaires. We used multivariate logistic regression to estimate the odds ratios (ORs) and 95% Confidence Intervals (CI) for each carbohydrate exposure in relation to healthy aging. A total of 3,731 NHS participants met our criteria for healthy aging. Refined carbohydrate intake was associated with lower odds of healthy aging (ORs[95% CIs] per 5%-calorie increase: 0.94[0.90, 0.97]). Conversely, intakes of carbohydrates from whole grains, fruits, and vegetables were associated with 2% to 6% higher odds of healthy aging for every 1%-calorie increase. Additionally, intakes of total fiber, fruit fiber, vegetable fiber and cereal fiber were associated with 12% to 25% higher odds of healthy aging per 1%-calorie increment. Lastly, the dietary GI was associated with lower odds of healthy aging. In conclusion, intakes of high-quality carbohydrate from fruits, vegetables, whole grains, and dietary fiber in middle adulthood were favorably associated with healthy aging in a large cohort of women, whereas refined carbohydrate intake was unfavorably associated with healthy aging.

